# Supplementary material for: The impact of wartime conflict on the mental health problems of women in the conflict-hit population in Woldia, Ethiopia
Source: PLoS One. 2025 Jun 9;20(6):e0322047. doi: 10.1371/journal.pone.0322047 (PMC12148197; doi:10.1371/journal.pone.0322047)
Supplement: Attachment — Submitted filename: PONE-D-24-23457.pdf [file pone.0322047.s002.pdf]

PLOS ONE

The impact of wartime conflict on mental health problems of women in conflict-hit population in Woldia, Ethiopia

--Manuscript Draft--

**Manuscript Number:**	PONE-D-24-23457
**Article Type:**	Research Article
**Full Title:**	The impact of wartime conflict on mental health problems of women in conflict-hit population in Woldia, Ethiopia
**Short Title:**	wartime conflict on mental health problems
**Corresponding Author:**	Negussie Deyessa<br>University of Rwanda College of Medicine and Health Sciences<br>Addis Ababa, ETHIOPIA
**Keywords:**	Common mental Illness,<br>Depression,<br>Generalized anxiety disorder,<br>Post-traumatic Stress Disorder,<br>Wartime conflict,<br>Women in Marriage,<br>Ethiopia.
**Abstract:**	Background<br><br>Common mental health problems are of significant public health importance, with severe social and economic impacts that adversely affect individuals' quality of life. The burden of these problems may worsen during wartime. This study aims to assess the prevalence of war-related common mental health problems, including depression, anxiety disorder, phobia, and post-traumatic stress disorder (PTSD), among women in the Woldia district, Amhara, Ethiopia.<br><br>Methods<br><br>A community-based cross-sectional survey was conducted from February to March 2023, involving 1,492 women selected from five kebeles using systematic sampling. The study used the Patient Health Questionnaire-9, Generalized Anxiety Disorder scale, and PTSD Checklist-5 to assess common mental health disorders. In addition to descriptive analysis, the study employed binary and multivariable analysis to evaluate socio-demographic correlates and the presence of comorbidity for each mental disorder.<br><br>Result<br><br>Almost half of the women exhibited major symptoms of common mental health problems, with about 33% experiencing comorbidity of two or more disorders. Depressive symptoms were more prevalent among older, single women and those with spouses using khat, whereas higher wealth and strong social support were protective factors. Similar risk and protective patterns were observed for generalized anxiety disorder (GAD) and post-traumatic stress disorder (PTSD). Older age, single status, and moderate income increased risk, while a higher wealth index and stronger social support provided some protection.<br><br>Conclusion<br><br>The findings from Woldia reveal a severe mental health crisis among women post-wartime, with elevated levels of depression, anxiety, and PTSD far exceeding global averages. This crisis jeopardizes the well-being of women and has far-reaching implications for families and communities, necessitating an urgent and multi-faceted approach to address both risk and protective factors identified in the study.
**Order of Authors:**	Negussie Deyessa

Powered by Editorial Manager® and ProduXion Manager® from Aries Systems Corporation

	Kehali Getahun, MD
	Ruth Filimona
	Abraraw Tadesse, MD
**Additional Information:**	
**Question**	**Response**
**Financial Disclosure**<br><br>Enter a financial disclosure statement that describes the sources of funding for the work included in this submission. Review the submission guidelines for detailed requirements. View published research articles from *PLOS ONE* for specific examples.<br><br>This statement is required for submission and **will appear in the published article** if the submission is accepted. Please make sure it is accurate.<br><br>**Funded studies**<br>Enter a statement with the following details:<br>• Initials of the authors who received each award<br>• Grant numbers awarded to each author<br>• The full name of each funder<br>• URL of each funder website<br>• Did the sponsors or funders play any role in the study design, data collection and analysis, decision to publish, or preparation of the manuscript?<br><br>Did you receive funding for this work?	The author(s) received no specific funding for this work.
**Competing Interests**<br><br>Use the instructions below to enter a competing interest statement for this submission. On behalf of all authors, disclose any competing interests that could be perceived to bias this work—acknowledging all financial support and any other relevant financial or non-financial competing interests.<br><br>This statement is required for submission	The authors have declared that no competing interests exist.

and **will appear in the published article** if the submission is accepted. Please make sure it is accurate and that any funding sources listed in your Funding Information later in the submission form are also declared in your Financial Disclosure statement.<br><br>View published research articles from *PLOS ONE* for specific examples.<br><br>**NO authors have competing interests**<br><br>Enter: *The authors have declared that no competing interests exist.*<br><br>**Authors with competing interests**<br><br>Enter competing interest details beginning with this statement:<br><br>*I have read the journal's policy and the authors of this manuscript have the following competing interests: [insert competing interests here]*<br><br>* typeset	
**Ethics Statement**<br><br>Enter an ethics statement for this submission. This statement is required if the study involved:<br><br>• Human participants<br>• Human specimens or tissue<br>• Vertebrate animals or cephalopods<br>• Vertebrate embryos or tissues<br>• Field research<br><br>Write "N/A" if the submission does not require an ethics statement.<br><br>General guidance is provided below. Consult the submission guidelines for detailed instructions. **Make sure that all information entered here is included in the Methods section of the manuscript.**	The study received ethical approval from the Institutional Review Board (IRB) of the Ethiopian Public Health Association, with a reference number of EPHA/OG/279/23 given on January 5, 2023. We collected the data by receiving informed consent from local leaders and individuals participating in the study with proper privacy and confidentiality, as in the WHO guide for collecting data on violence against women

Powered by Editorial Manager® and ProduXion Manager® from Aries Systems Corporation

**Format for specific study types**<br><br>**Human Subject Research (involving human participants and/or tissue)**<br>• Give the name of the institutional review board or ethics committee that approved the study<br>• Include the approval number and/or a statement indicating approval of this research<br>• Indicate the form of consent obtained (written/oral) or the reason that consent was not obtained (e.g. the data were analyzed anonymously)<br><br>**Animal Research (involving vertebrate animals, embryos or tissues)**<br>• Provide the name of the Institutional Animal Care and Use Committee (IACUC) or other relevant ethics board that reviewed the study protocol, and indicate whether they approved this research or granted a formal waiver of ethical approval<br>• Include an approval number if one was obtained<br>• If the study involved *non-human primates*, add *additional details* about animal welfare and steps taken to ameliorate suffering<br>• If anesthesia, euthanasia, or any kind of animal sacrifice is part of the study, include briefly which substances and/or methods were applied<br><br>**Field Research**<br><br>Include the following details if this study involves the collection of plant, animal, or other materials from a natural setting:<br>• Field permit number<br>• Name of the institution or relevant body that granted permission	
**Data Availability**<br><br>Authors are required to make all data underlying the findings described fully available, without restriction, and from the time of publication. PLOS allows rare exceptions to address legal and ethical concerns. See the PLOS Data Policy and FAQ for detailed information.	Yes - all data are fully available without restriction

A Data Availability Statement describing where the data can be found is required at submission. Your answers to this question constitute the Data Availability Statement and **will be published in the article**, if accepted.<br><br>**Important:** Stating 'data available on request from the author' is not sufficient. If your data are only available upon request, select 'No' for the first question and explain your exceptional situation in the text box.<br><br>Do the authors confirm that all data underlying the findings described in their manuscript are fully available without restriction?	
**Describe where the data may be found in full sentences. If you are copying our sample text, replace any instances of XXX with the appropriate details.**<br><br>• If the data are **held or will be held in a public repository**, include URLs, accession numbers or DOIs. If this information will only be available after acceptance, indicate this by ticking the box below. For example: *All XXX files are available from the XXX database (accession number(s) XXX, XXX.*).<br>• If the data are all contained **within the manuscript and/or Supporting Information files**, enter the following: *All relevant data are within the manuscript and its Supporting Information files.*<br>• If neither of these applies but you are able to provide **details of access elsewhere**, with or without limitations, please do so. For example:<br><br>*Data cannot be shared publicly because of [XXX]. Data are available from the XXX Institutional Data Access / Ethics Committee (contact via XXX) for researchers who meet the criteria for access to confidential data.*<br><br>*The data underlying the results presented in the study are available from (include the name of the third party*	All relevant data are within the manuscript and its Supporting Information files.

and contact information or URL).<br>• This text is appropriate if the data are owned by a third party and authors do not have permission to share the data.<br><br>* typeset	
Additional data availability information:	

Manuscript
Click here to access/download;Manuscript;The impact of conflict, June 09 2024.docx

The impact of wartime conflict on mental health problems of women in conflict-hit population in Woldia, Ethiopia

Negussie Deyessa[1*a], Kehali Getahun[2b], Ruth Filimona[3b], Abraraw Tadesse[4c]

Affiliation of investigators

[1]Department of Epidemiology and Biostatistics; School of Public Health, College of Medicine and Health Sciences, University of Rwanda [ND= negdaysun@gmail.com]

[2]School of Medicine, College of Health Sciences, Addis Ababa University, [KG = kehaliye@gmail.com]

[3]Department of Emergency, Gynecology and Obstetrics, Gandhi Memorial Hospital, Addis Ababa, Ethiopia, [RF = ruthfili@gmail.com].

[4]Department of Communication, Amhara Regional Health Bureau, Bahirdar, Ethiopia, [RF = abrarawtadesse2121@gmail.com].

Corresponding Author*

Address: P. O. Box 3286, **KG 175 Street, Kigali**, Rwanda; Telephone +250911793762811; and Email: negdaysun@gmail.com

Abstract

Background: Common mental health problems are of significant public health importance, with severe social and economic impacts that adversely affect individuals' quality of life. The burden of these problems may worsen during wartime. This study aims to assess the prevalence of war-related common mental health problems, including depression, anxiety disorder, phobia, and post-traumatic stress disorder (PTSD), among women in the Woldia district, Amhara, Ethiopia.

Methods: A community-based cross-sectional survey was conducted from February to March 2023, involving 1,492 women selected from five kebeles using systematic sampling. The study used the Patient Health Questionnaire-9, Generalized Anxiety Disorder scale, and PTSD Checklist-5 to assess common mental health disorders. In addition to descriptive analysis, the study employed binary and multivariable analysis to evaluate socio-demographic correlates and the presence of comorbidity for each mental disorder.

Result: Almost half of the women exhibited major symptoms of common mental health problems, with about 33% experiencing comorbidity of two or more disorders. Depressive symptoms were more prevalent among older, single women and those with spouses using khat, whereas higher wealth and strong social support were protective factors. Similar risk and protective patterns were observed for generalized anxiety disorder (GAD) and post-traumatic stress disorder (PTSD). Older age, single status, and moderate income increased risk, while a higher wealth index and stronger social support provided some protection.

Conclusion: The findings from Woldia reveal a severe mental health crisis among women post-wartime, with elevated levels of depression, anxiety, and PTSD far exceeding global averages. This crisis jeopardizes the well-being of women and has far-reaching implications for families

and communities, necessitating an urgent and multi-faceted approach to address both risk and protective factors identified in the study.

Keywords: Common Mental Illness, Depression, Generalized anxiety disorder, Post-traumatic Stress Disorder, Wartime conflict, Women in Marriage, Ethiopia.

Introduction

In developing countries, common mental health problems, comprising depression, anxiety disorders, and post-traumatic stress disorders, remain some of the most common and debilitating mental health problems, having severe public health, social, and economic impacts adversely affecting individuals' quality of life [1]. The triggers and manifestations of depression in these settings often intertwine with the unique socioeconomic and cultural factors inherent to these nations [2]. Generalized Anxiety Disorder (GAD) and Post-Traumatic Stress Disorder (PTSD) are also prevalent mental health concerns in developing countries [3]. GAD often manifests in individuals as a chronic, excessive worry about everyday events, exacerbated by the unstable socio-political environments, food insecurity, and financial uncertainties common in many nations[3]. On the other hand, PTSD often stems from direct exposure to traumatic events, which can be too familiar in developing countries due to conflicts, wars, natural disasters, or personal traumas like assault and abuse [4].

The global burden of depression, generalized anxiety disorders (GAD), and post-traumatic stress disorders (PTSD) is substantial and has profound implications for individuals, communities, and health systems worldwide [5]. Depression, the typical psychological condition, affects about 121 million people worldwide, and it is the leading cause of disability as measured by Years Lived with Disability (YLDs) and the fourth top contributor to the global disease burden [6]. GAD, characterized by persistent and excessive worry, further amplifies this burden, affecting many individuals across diverse socio-cultural backgrounds [3]. PTSD, arising from exposure to traumatic events, including wars, natural disasters, and personal assaults, has long-term repercussions on the mental health of survivors. In many parts of the world, especially in low-resource settings, the limited availability of mental health services, combined with societal

stigma, exacerbates the impact of these disorders, highlighting the urgent need for global strategies and interventions to address this mounting challenge [7].

In developing countries, several key determinants influence the mental health landscape. Pervasive poverty and associated challenges, such as food insecurity and limited access to necessities, often form the bedrock for mental distress [8]. The constant strains of political instability, civil unrest, or warfare create environments where trauma and subsequent mental disorders, like PTSD, flourish [9]. Infectious diseases, including HIV/AIDS and malaria, pose direct health threats and contribute to psychological distress and societal disruption [10]. Cultural and societal stigmas related to mental health often hinder individuals from seeking timely help, while the shortage of mental health professionals and infrastructure makes access to care challenging [11].

Wartime profoundly affects the mental health of populations, particularly women in developing countries. The mechanisms through which wartime exacerbates mental health issues in these women are multi-faceted, but to mention some, direct exposure to violence in which women witness or become victims of brutal acts of violence. Witnessing such violence can lead to trauma, manifesting as PTSD or other stress-related disorders [12, 13]. Conflict situations often involve a heightened risk of sexual assault, rape, and other gender-based violence. These traumatic events have significant psychological repercussions, leading to feelings of shame, guilt, depression, anxiety, and PTSD [12]. Many women lose family members due to direct conflict or related factors like disease or starvation. The grief and trauma associated with such loss can be overwhelming [14]. War often forces populations to flee their homes, leading to internal displacement or refuge in other countries. This dislocation from familiar settings and the hardships of refugee life can lead to feelings of rootlessness, anxiety, and depression [15].

Wartime often disrupts regular means of livelihood. Women, especially those heading households, face the pressure of providing for their families under constrained circumstances, leading to chronic stress. The traditional roles and societal structures can be upended during the war, leading to familial and societal expectations changes, which can further add to women's stress and mental health challenges [16]. Already constrained healthcare resources in developing countries become even more limited during the conflict, making it challenging to address physical and mental health needs. Women of reproductive age or those with children face added challenges. War trauma can complicate pregnancies, and the stress of ensuring a child's safety and well-being in such an environment can be immense. Even after the active conflict ends, the traumatic memories and the socioeconomic repercussions persist, leading to chronic mental health issues [17].

In the North Ethiopian conflict context, understanding the ripple effects of war on the population, especially women, is vital. War often creates an environment where social norms and structures are disrupted, leading to increased incidents like common mental health disorders. Furthermore, the traumatic experiences from such conditions, compounded by the general stresses of war, can significantly impact mental health. Women, already vulnerable due to the societal dynamics of the conflict region, may find themselves grappling with a range of mental health challenges as a direct outcome. Research in this area sheds light on the immediate effects but also aids in understanding the intricate relationship between war-induced incidents and mental health repercussions. Therefore, this research aims to assess the magnitude of war-related common mental health problems, depression, anxiety disorder, phobia, and post-traumatic stress disorder among women in Woldia district, Amhara, Ethiopia.

Methods

Setting**:** The study took place in Woldia woreda, which encompasses ten kebeles that experienced severe disruption, leading to significant humanitarian challenges for three months in 2021, with limited access to food, clean water, and sanitation. Woldia is a town and the capital of the North Wollo Zone in northern Ethiopia [18]. It is located 525 km north of Addis Ababa and 360 km from Bahir Dar, the capital of the Amhara Regional State, situated north of Dessie and southeast of Lalibela. According to the 2007 Ethiopia national census, the projected population size of the town was 79,667 in 2010, with 49.4% being women [19]. The health institution profile of the city includes one public hospital, two public health centers, and several private pharmacies and clinics providing healthcare services to the community [18].

Study Design: - The study used a cross-sectional survey to measure conflict-related common mental disorders among women in the Woldia district from February to March 2023.

Target population and sampling plan: - The study included women in North Wollo zones of Amhara, where the conflict escalated, specifically women of 18 or more years who were in place during the war, living in Woldia town or a rural community. However, it excluded women who were unavailable in the district during wartime, unable to communicate, chronically sick, and suffering from pain.

Sample size and sampling: We determined the sample size for the study based on previous evidence suggesting that 4.8% of women of reproductive age without IPV would have common mental disorders, primarily depression[20]. Assuming that women in conflict-affected areas with a lack of medication, social support, and exposure to IPV or GBV would experience a two-fold increase, we used a 95% CI, 80% power, and a design effect of 1.5. After adding 10% to compensate for possible non-response, we included 1,492 women in the study. We employed multistage sampling for the survey. In the first phase, we selected five of the ten available

clusters using simple random sampling. In the second stage, we chose households through systematic sampling, distributing the sample size to each kebele proportionate to the population size of households, taking households as the sampling unit. We created a sampling frame for each household with at least one eligible woman, assigning sequential numbers in a map sketch. After determining the sampling interval, data collectors randomly identified the first household as a starting point. They then added the sampling interval as a fixed increment, continuing until all chosen study subjects were interviewed. Enumerators randomly selected only one eligible woman for an interview in households with two or more eligible women.

Data Collection and Measurement: The study used a validated and standard questionnaire to measure socio-demographics, life experiences during wartime, and common mental illnesses. We developed the questionnaire in the local language, adapting it from previous versions of the instruments tested in other research. The study recruited and trained eighteen data collectors, two supervisors, and a coordinator for three days on the contents of the questionnaire. We pre-tested the questionnaire during the training of data collectors to amend any mismatches in local contextual linguistics. Enumerators collected the data using Open Data Kit, and we exported it into SPSS for Windows for cleaning and analysis.

The study included socio-demographic characteristics of the women, such as age, sex, current marital status, life experience during wartime, and experiences related to threatening conditions. We measured the psychosocial problems of women using standard tools, including PHQ-9, the General Anxiety Disorder-7 (GAD-7), and the PTSD-Checklist for DSM-5 (PCL-5). The study also used the WHO-5 Well-being Index to supplement the assessment of the mental health status of the women.

The PHQ-9 is a nine-item questionnaire measuring symptoms of depression. It is a well-established 9-item screening tool to assess symptoms of depression [21]. The module asks respondents to indicate how frequently they experienced depressive symptoms in the past two weeks, rating these on a scale of zero (never) to three (every day). The PHQ-9 is scored by summing up the nine responses, leading to a scale ranging between 0 to 27. As a severity measure, 5, 10, 15, and 20 cutoff points represent the thresholds for mild, moderate, moderately severe, and severe depression, respectively. If a single screening cutoff point is required, it is currently recommended to use a PHQ-9 score of 10 or greater, which has a high sensitivity and specificity of major depression [21].

The study also used the Generalized Anxiety Disorder (GAD-7), a seven-item tool assessing generalized anxiety disorder severity. Respondents rate symptoms from the past two weeks, with scores ranging from 0 to 3 based on frequency. It is a screening and severity measure for primary anxiety disorders, including GAD, Panic Disorder, Social Phobia, and PTSD. Validated in various settings, a study among Ethiopian University students [22] categorized GAD-7 scores: 0-4 as none, 5-9 as mild, 10-14 as moderate, and 15+ as severe anxiety [23]. Additionally, a single question evaluated functional difficulty related to anxiety.

Furthermore, the PTSD-PCL-5 is a 20-item measure that assesses the 20 DSM-5 symptoms of PTSD. The PCL-5 has a variety of purposes, including screening individuals for PTSD and making a provisional PTSD diagnosis. A total symptom severity score ranges from 0-80 and can be obtained by summing the scores for each of the twenty items. Research suggests a PCL-5 cutoff score between 31 and 33 indicates probable PTSD across samples. A provisional PTSD diagnosis can be made by treating each item rated as two “Moderately” or higher as a symptom

endorsed, then following the *DSM-5* diagnostic rule, which requires at least 1 B item (questions 1-5), 1 C item (questions 6-7), 2 D items (questions 8-14), and 2 E items (questions 15-20) [24].

The WHO-5 Well-Being is a five-item questionnaire that measures the general well-being of an individual in terms of a short form of quality of life. It has five item questions with six six-point Likert scales, ranging from '0 = total absence' to '5 = strong feeling' of well-being. The algorithm of the instrument includes summing the total score and multiplying the result by four to obtain a percentage score. The higher the score, the better an individual's well-being. WHO-5 well-being questionnaire was used and validated in Ethiopia among adults to see for perceived overall change [25].

Confounding and effect-modifying variables

Possible confounding variables in this analysis include a range of women's social, economic, and war-related experiences and the substance use characteristics of their spouses. Key confounding variables that will be measured include participants' age and sex, social status, household income, social networks, women's experience in the war, and spousal substance use. Furthermore, the study will measure study subjects' socio-emotional competencies measured using general self-efficacy, and their coping skills will be assessed using the brief COPE score. We examined the impact of socio-demographic and other factors that interacted with a range of effect-modifying variables. These variables were specified in advance to ensure a significant impact is deliberately highlighted.

Data Analysis

Data from the field will be directly entered into an Excel spreadsheet on a computer and exported into SPSS for Windows. After cleaning and transforming them into meaningful variables, we assessed them for simple descriptive analysis. We further analyzed war-inflicted common mental health problems descriptively to find their magnitude in the sample and the population using a point estimate of the proportion and its 95% confidence interval, respectively.

The study used logistic regression to assess the association between socio-demographic, war-related disruptions against common mental health problems. Each possible explanatory variable, including socio-demographic characteristics, war-related experiences, and other factors, was assessed for association first with each common mental illness status outcome variable, including depression, anxiety, and PTSD. We also evaluated each explanatory variable for association with the primary explanatory variable, war-related experiences. Explanatory variables having an association or borderline association with both the outcome and the explanatory variables were included in the analysis model between explanatory and outcome variables after assessing for multicollinearity.

Following adjusting for potential confounders in the multivariable logistic regression analysis, we further assessed for their confounding, mediation, or interaction effect on the possible confounders in the model by including them one by one at a time to realize. Finally, we disclosed the model describing mediators and interacting variables as analysis findings.

Ethical consideration

The study received ethical approval from the Institutional Review Board (IRB) of the Ethiopian Public Health Association, with a reference number EPHA/ OG/ 279/ 23 given on January 05,

2023. We collected the data by receiving informed consent from local leaders and individuals participating in the study with proper privacy and confidentiality as in the WHO guide for collecting data on violence against women [26].

Results

The research encompassed 1,505 female participants from five kebeles within Woldia city. A prominent segment, accounting for 41.7%, fell into the age group between 25 to 34 years, with a mean age of 36.2 $\pm$ 10.2, spanning an age range of 18 to 60 years. Over two-thirds of these participants were in a marital union. Regarding educational background, 25% of the participants reported no formal education, while approximately 10% attained tertiary-level education. A significant 87.2% were religiously identified as Orthodox Christian adherents and 10.7% as Muslims. A predominant portion of these women was engaged in non-remunerative roles. In contrast, a minor segment, approximating ten per cent, was employed in either governmental or private sectors, as detailed in Table 1.

Table 1. Socio-demographic characteristics of women who were in Woldia during the wartime, Woldia, north Ethiopia, April 2023

Characteristics	Frequency	Percent
Age group		
18-24 yrs	108	7.2
25-34 yrs	623	41.4
35-44 yrs	418	27.8
45-54 yrs	235	15.0
55 yrs or more	131	8.7
Mean + SD	36.2 + 10.2	
Marital status		
Married	1087	72.2
Currently single	51	3.4

Divorced or separated.	215	14.3
Widowed	152	10.1
Educational status		
Not educated	375	24.9
Elementary [0-8]	529	35.1
Secondary [9-12]	453	30.1
Tertiary [BSc or more]	148	9.8
Religion		
Orthodox	1312	87.2
Muslim	161	10.7
Protestant	32	2,1
Occupation		
Unpaid work	1038	79.3
Employee	138	10.5
Trading	111	8.5
Other.	22	1.7

Economically, the study revealed that over 37% of participants reported a monthly income below 1,500 Ethiopian Birr, with two-thirds earning less than 3,000 Ethiopian Birr (equivalent to $60). When categorizing participants based on household assets, 26.5% were classified within the lower wealth index, while half belonged to the moderate category. Regarding self-perceived economic status relative to their neighbours, approximately 55% perceived themselves as in a lower bracket, and 39.3% viewed themselves as moderately positioned. Concerning community social interactions, about 41% and 51% of participants perceived themselves as having a lower and moderate social status, respectively. Utilizing the Oslo-social support index as a metric, 50%

of participants scored in the lower range, whereas a mere 15% achieved a strong score. Furthermore, 11% of participants faced economic challenges attributed to their spouses' alcohol consumption, and about 4% encountered economic issues linked to their spouses' Khat chewing habits, as illustrated in Table 2.

Table 2. Current reproductive health, Social, and economic related characteristics of women who were in Woldia during the wartime, Woldia, north Ethiopia, April 2023

Characteristics	frequency	Percent
Monthly income [eth. Birr]		
Below 1500 Birr	564	37.7
1500 to 2999 Birr	373	24.9
Three thousand Birr or more.	560	37.4
Wealth index		
Lower wealth	404	26.8
Medium wealth	753	50.0
Higher wealth.	348	23.1
Perceived economic status.		
Higher	84	5.6
Moderate	591	39.3
Lower	830	55.1
Perceived social status.		
Higher	121	8.0
Moderate	768	51.0
Lower	616	40.9
Oslo-social support index		

Poor	734	48.8
Moderate	547	36.3
Strong	223	14.8
Spousal alcohol-related problem		
No	1340	89.0
Yes	165	11.0
Spousal Khat-related problem		
No	1444	95.9
Yes	61	4.1

The magnitude of common mental health problems in the women population in Woldia during the war was evaluated as follows. The results indicate that 60.9% [95% CI: 58.5, 63.4] of women felt their lives lacked meaning. Concurrently, 32.2% [95% CI: 29.8, 34.5] exhibited moderate to severe depressive symptoms, and 5.0% [95% CI: 3.9, 6.1] presented with severe forms of depressive manifestations. When measured against the criteria for generalized anxiety disorder (GAD), 22.7% [95% CI: 20.5, 24.8] demonstrated to have symptoms consistent with moderate GAD, while 10.5% [95% CI: 8.9, 12.0] met the criteria for a severe form of GAD. In terms of post-traumatic stress disorder (PTSD) symptomatology, 44.3% [95% CI: 41.8, 46.8] of women exhibited to have symptoms of the syndrome. Notably, within this segment, 20.9% displayed one or two major PTSD symptoms, and 23.4% manifested three or four major symptoms of the syndrome. Lastly, more than half of the women displayed at least one of the common mental health disorders mentioned above, of which 33.0% [95% CI: 30.5, 35.3] of the women exhibited to have a co-occurrence of two or more of these mental disorders, as in (Table 3).

Table 3. The magnitude of common mental health problems among women who were in Woldia during the wartime, Woldia, north Ethiopia, April 2023

Characteristics	frequency	Percent (95% CI)
WHO-wellbeing [lower score]	917	60.9% [58.5, 63.4]
PHQ-9 based scoring.		
None		
Moderate [10-19]	484	32.2 [29.8, 34.5]
Severe depression [20 or more]	75	5.0 [3.9, 6.1]
Generalized anxiety disorder		
None		
Moderate anxiety	341	22.7 [20.5, 24.8]
Severe anxiety	158	10.5 [8.9, 12.0]
Presence of PTSD		
None		
Present	667	44.3 [41.8, 46.8]
Symptoms of PTSD		
Intrusion symptoms	568	37.7 [35.3, 40.2]
Avoidance of stimuli	520	34.6 [32.1, 37.0]
Alteration of cognition and mood	298	19.8 [17.8, 21.8]
Alteration in arousal and reactivity	315	20.9 [18.9, 23.0]
Number of Symptoms of PTSD		
1-2 major symptoms	315	20.9 [19.9, 23.0]
3-4 major symptoms	352	23.4 [21.3, 25.5]
Comorbidity		

None	751	49.9 [47.4, 52.4]
Only one disorder	258	17.1 [15.2, 19.0]
Two or more disorders	496	33.0 [30.5, 35.3]

Determinants of common mental illness

The study broadly indicated that elements like marital status, the mother's job, family income, perceived social standing and issues related to a spouse's use of alcohol and khat were commonly associated with depression, generalized anxiety disorder, and post-traumatic stress disorder. These factors were integrated into the models for these prevalent mental health issues. Moreover, age and perceived economic status had a specific association with depression. Religion, age at the time of marriage, and perceived economic status were related to generalized anxiety disorder. A woman's number of children was explicitly linked to post-traumatic stress disorder. These specialized factors were also included in the respective models for each mental health condition. [Table 4-5]

After accounting for various factors, we found that depressive symptoms were more common among older women, single women, and women whose spouses have issues related to khat chewing. On the other hand, women in trading jobs, those with a medium to high wealth index, and those who perceive themselves as having moderate to high social and economic status, as well as those with moderate to strong social support (measured by the Oslo-social support index), were less likely to report depression.

In the case of generalized anxiety disorder, older age, single marital status, and moderate monthly income were positively associated with the condition. Conversely, having a medium to

high wealth index and moderate to strong social support (again, measured by the Oslo-social support index) reduced the likelihood of experiencing generalized anxiety disorder.

Lastly, for post-traumatic stress disorder (PTSD), the condition was more common among older women, those who are currently single or widowed, women with moderate monthly incomes, and women with moderate levels of social support. However, women in trading occupations, those with a medium to high wealth index, those who perceive their social status as moderate, and those with strong social support were less likely to have PTSD, [Table 6 and 7].

Table 4. Socio-demographic correlates of common mental health problems of women who were in Woldia during the wartime, Woldia, north Ethiopia, April 2023

Characteristics	Sample	Depression (PHQ-9 ≥ 10) n (%)	Crude OR (95% CI)	Anxiety Dis. (Mod-Severe) n (%)	Crude OR (95% CI)	PTSD n (%)	Crude OR (95% CI)
Age group							
18-24 yrs	108	32 (29.6)	1.00	35 (32.4)	1.00	37 (34.3)	1.00
25-34 yrs	623	222 (35.6)	1.32 (0.84, 2.05)	200 (32.1)	0.99 (0.64, 1.52)	186 (29.9)	0.82 (0.53, 1.26)
35-44 yrs	418	152 (36.4)	1.36 (0.86, 2.15)	138 (33.0)	1.03 (0.65, 1.62)	150 (35.9)	1.07 (0.69, 1.68)
45-54 yrs	235	92 (40.9)	1.64 (1.01, 2.69)	76 (33.8)	1.06 (0.65, 1.73)	81 (36.0)	1.08 (0.67, 1.75)
55 yrs or more	131	61 (46.6)	2.07 (1.21, 3.54)	50 (38.2)	1.29 (0.75, 2.20)	54 (41.2)	1.35 (0.79, 2.28)
Marital status							
Married	1087	366 (33.7)	1.00	340 (31.3)	1.00	326 (30.0)	1.00
Currently single	51	29 (56.9)	2.59 (1.47, 4.58)	28 (54.6)	2.68 (1.52, 4.71)	38 (74.5)	6.82 (3.59, 12.9)
Divorced or separated.	215	95 (44.2)	1.56 (1.16, 2.10)	75 (34.9)	1.18 (0.87, 1.60)	75 (34.9)	1.25 (0.92, 1.70)
Widowed	152	69 (45.4)	1.64 (1.16, 2.31)	56 (36.8)	1.28 (0.90, 1.83)	69 (45.4)	1.94 (1.38, 2.74)
Educational status							
Not educated	375	170 (45.3)	1.00	133 (35.5)	1.00	146 (38.9)	1.00
Elementary [0-8]	529	194 (36.7)	0.70 (0.53, 0.91)	193 (36.5)	1.05 (0.79, 1.38)	180 (34.0)	0.81 (0.62, 1.07)
Secondary [9-12]	453	161 (35.5)	0.67 (0.50, 0.88)	140 (30.9)	0.81 (0.61, 1.09)	146 (32.2)	0.75 (0.56, 0.99)
Tertiary [BSc or more]	148	34 (23.0)	0.36 (0.23, 0.56)	33 (22.3)	0.52 (0.34, 0.81)	36 (24.3)	0.50 (0.33, 0.77)
Religion							
Orthodox	1312	496 (37.8)	1.00	448 (34.1)	1.00	441 (33.6)	1.00
Muslim	161	52 (32.3)	0.79 (0.55, 1.11)	41 (25.5)	0.66 (0.45, 0.96)	56 (34.8)	1.05 (0.75, 1.49)
Protestant	32	11 (34.4)	0.86 (0.41, 1.80)	10 (31.3)	0.88 (0.41, 1.87)	11 (34.4)	1.04 (0.49, 2.17)
Occupation							
Unpaid work	1038	422 (40.7)	1.00	370 (35.6)	1.00	383 (36.9)	1.00
Employee	138	30 (21.7)	0.41 (0.27, 0.62)	26 (18.8)	0.42 (0.27, 0.65)	32 (23.2)	0.52 (0.34, 0.78)
Trading	111	30 (27.0)	0.54 (0.35, 0.84)	32 (28.8)	0.73 (0.48, 1.12)	28 (25.2)	0.58 (0.37, 0.90)
Other.	22	14 (63.6)	2.56 (1.06, 6.14)	11 (50.0)	1.81 (0.78, 4.20)	12 (54.5)	2.05 (0.88, 4.80)
Monthly income [eth. Birr]							
Below 1500 Birr	564	222 (39.4)	1.00	191 (33.9)	1.00	207 (36.7)	1.00
1500 to 2999 Birr	373	185 (49.6)	1.52 (1.16, 1.97)	183 (49.1)	1.88 (1.44, 2.46)	175 (46.9)	1.52 (1.17, 1.99)
3000 Birr or more.	560	148 (26.4)	0.55 (0.43, 0.71)	125 (22.3)	0.56 (0.43, 0.73)	124 (22.1)	0.49 (0.38, 0.64)

Table 5. Current reproductive health, Social, and economic related correlates of common mental health problem of women who were in Woldia during the wartime, Woldia, north Ethiopia, April 2023

Characteristics	Sample	Depression (PHQ-9 ≥ 10) n (%)	Crude OR (95% CI)	Anxiety Dis. (Mod-severe) n (%)	Crude OR (95% CI)	PTSD n (%)	Crude OR (95% CI)
Age at marriage							
< 15 yrs	97	28 (28.9)	1.00	23 (23.7)	1.00	34 (35.1)	1.00
15-17 yrs	317	123 (38.8)	1.56 (0.95, 2.56)	99 (31.2)	1.46 (0.87, 2.47)	106 (33.4)	0.93 (0.58, 1.50)
18 yrs. or more.	1070	397 (37.1)	1.45 (0.92, 2.29)	369 (34.5)	1.69 (1.04, 2.75)	359 (33.6)	0.94 (0.61, 1.45)
Number of children							
No child	117	47 (40.2)	1.00	41 (35.0)	1.00	54 (46.2)	1.00
1-2 children	849	315 (37.1)	0.88 (0.59, 1.30)	282 (33.2)	0.92 (0.61, 1.38)	284 (33.5)	0.59 (0.40, 0.87)
3 or more	539	197 (36.5)	0.86 (0.57, 1.29)	176 (32.7)	0.90 (0.59, 1.37)	170 (31.5)	0.54 (0.36, 0.81)
Wealth index							
Lower wealth	404	231 (57.2)	1.00	213 (52.7)	1.00	196 (48.5)	1.00
Medium wealth	753	252 (33.5)	0.38 (0.29, 0.48)	222 (29.5)	0.38 (0.29, 0.48)	238 (31.7)	0.49 (0.39, 0.83)
Higher wealth	348	76 (21.8)	0.21 (0.15, 0.29)	64 (18.4)	0.20 (0.15, 0.28)	73 (21.0)	0.62 (0.49, 0.77)
Perceived economic status.							
Higher	84	15 (17.9)	0.28 (0.16, 0.50)	12 (14.3)	0.27 (0.14, 0.50)	20 (23.8)	0.49 (0.29, 2.33)
Moderate	591	182 (30.8)	0.56 (0.46, 0.72)	168 (28.4)	0.64 (0.51, 0.80)	166 (28.1)	0.74 (0.50, 1.08)
Lower	830	362 (43.6)	1.00	319 (38.4)	1.00	322 (38.8)	1.00
Perceived social status.							
Higher	121	26 (21.5)	0.29 (0.19, 0.47)	23 (19.0)	0.33 (0.20, 0.53)	33 (27.3)	0.47 (0.30, 0.72)
Moderate	768	236 (30.7)	0.48 (0.38, 0.59)	219 (28.5)	0.56 (0.45, 0.70)	200 (26.0)	0.44 (0.35, 0.55)
Lower	616	297 (48.2)	1.00	257 (41.7)	1.00	275 (44.6)	1.00
Oslo-social support index							
Poor	734	347 (47.3)	1.00	301 (41.0)	1.00	274 (37.3)	1.00
Moderate	547	173 (31.6)	0.52 (0.41, 0.65)	162 (29.6)	0.61 (0.48, 0.77)	188 (34.4)	0.88 (0.70, 1.11)
Strong	223	39 (17.5)	0.24 (0.16, 0.34)	36 (16.1)	0.28 (0.19, 0.41)	45 (20.2)	0.42 (0.30, 0.61)
Spousal alcohol-related prob.							
No	1340	478 (35.7)	1.00	428 (31.9)	1.00	448 (33.4)	1.00
Yes	165	81 (49.1)	1.74 (1.26, 2.41)	71 (43.0)	1.61 (1.16, 2.24)	60 (36.4)	1.14 (0.81, 1.59)
Spousal Khat-related problem	1444	527 (36.5)	1.00	471 (32.6)	1.00	483 (33.4)	1.00
No	61	32 (52.5)	1.92 (1.15, 3.21)	28 (45.9)	1.75 (1.05, 2.94)	25 (41.0)	1.38 (0.82, 2.33)
Yes							

Table 6. Socio-demographic correlates of common mental health problems of women who were in Woldia during the wartime, Woldia, north Ethiopia, April 2023

Characteristics	Depression [PHQ-9 score ≥ 10]		Generalized Anxiety Disorder (GAD) [Moderate or Sever GAD]		Post-traumatic stress disorder [PTSD]	
	Crude OR (95% CI)	Adjusted* OR (95% CI)	Crude OR (95% CI)	Adjusted** OR (95% CI)	Crude OR (95% CI)	Adjusted*** OR (95% CI)
Age group						
18-24 yrs	1.00	1.00	1.00	1.00	1.00	1.00
25-34 yrs	1.32 (0.84, 2.05)	2.03 (1.22, 3.39)	0.99 (0.64, 1.52)	1.24 (0.77, 1.99)	0.82 (0.53, 1.26)	1.13 (0.68, 1.88)
35-44 yrs	1.36 (0.86, 2.15)	2.44 (1.41, 4.25)	1.03 (0.65, 1.62)	1.51 (0.92, 2.50)	1.07 (0.69, 1.68)	2.10 (1.21, 3.66)
45-54 yrs	1.64 (1.01, 2.69)	3.51 (1.90, 6.47)	1.06 (0.65, 1.73)	1.71 (0.98, 3.00)	1.08 (0.67, 1.75)	2.40 (1.29, 4.47)
55 yrs or more	2.07 (1.21, 3.54)	3.10 (1.56, 6.16)	1.29 (0.75, 2.20)	2.32 (1.22, 4.41)	1.35 (0.79, 2.28)	2.03 (1.01, 4.07)
Marital status						
Married	1.00	1.00	1.00	1.00	1.00	1.00
Currently single	2.59 (1.47, 4.58)	2.39 (1.18, 4.83)	2.68 (1.52, 4.71)	2.36(1.16, 4.78)	6.82 (3.59, 12.9)	7.35 (3.14, 17.2)
Divorced or separated.	1.56 (1.16, 2.10)	1.17 (0.81, 1.69)	1.18 (0.87, 1.60)	0.95 (0.65, 1.39)	1.25 (0.92, 1.70)	0.90 (0.62, 1.30)
Widowed	1.64 (1.16, 2.31)	1.35 (0.86, 2.11)	1.28 (0.90, 1.83)	1.49 (0.96, 2.31)	1.94 (1.38, 2.74)	1.68 (1.08, 2.59)
Educational status						
Not educated	1.00	1.00	1.00	1.00	1.00	1.00
Elementary [0-8]	0.70 (0.53, 0.91)	0.97 (0.69, 1.37)	1.05 (0.79, 1.38)	1.10 (0.79, 1.52)	0.81 (0.62, 1.07)	1.09 (0.51, 1.44)
Secondary [9-12]	0.67 (0.50, 0.88)	1.03 (0.70, 1.51)	0.81 (0.61, 1.09)	0.92 (0.64, 1.34)	0.75 (0.56, 0.99)	1.13 (0.76, 1.67)
Tertiary [BSc or more]	0.36 (0.23, 0.56)	0.89 (0.49, 1.59)	0.52 (0.34, 0.81)	0.80 (0.47, 1.34)	0.50 (0.33, 0.77)	0.88 (0.48, 1.60)
Occupation						
Unpaid work	1.00	1.00	1.00	1.00	1.00	1.00
Employee	0.77 (0.46, 1.28)	0.77 (0.46, 1.28)	0.42 (0.27, 0.65)	1.05 (0.76, 1.47)	0.52 (0.34, 0.78)	0.86 (0.51, 1.44)
Trading	0.52 (0.32, 0.84)	0.52 (0.32, 0.84)	0.73 (0.48, 1.12)	0.82 (0.56, 1.20)	0.58 (0.37, 0.90)	0.61 (0.38, 0.99)
Other.	2.56 (0.97, 6.71)	2.56 (0.97, 6.71)	1.81 (0.78, 4.20)	0.72 (0.40, 1.29)	2.05 (0.88, 4.80)	2.16 (0.85, 5.48)
Religion						
Orthodox	1.00	= =	1.00	1.00	1.00	= =
Muslim	0.79 (0.55, 1.11)	= = = = = = = = =	0.66 (0.45, 0.96)	0.81 (0.52, 1.27)	1.05 (0.75, 1.49)	= = = = = = = = =
Protestant	0.86 (0.41, 1.80)	= = = = = = = = =	0.88 (0.41, 1.87)	1.47 (0.61, 3.51)	1.04 (0.49, 2.17)	= = = = = = = = =
Monthly income [eth. Birr]						
Below 1500 Birr	1.00	1.00	1.00	1.00	1.00	1.00
1500 to 2999 Birr	1.52 (1.16, 1.97)	1.55 (1.15, 2.09)	1.88 (1.44, 2.46)	1.84 (1.36, 2.49)	1.52 (1.17, 1.99)	1.40 (1.04, 1.88)
3000 Birr or more.	0.55 (0.43, 0.71)	1.07 (0.77, 1.48)	0.56 (0.43, 0.73)	0.91 (0.65, 1.28)	0.49 (0.38, 0.64)	0.80 (0.57, 1.11)

* Depression: Age grouped, Occupational status, educational status, Marital status, Monthly income, Household wealth, Oslo social support status, perceived economic position, perceived social position, presence of alcohol drinking problem, Presence of Khat chewing-related problem.

** Variable(s) entered on step 1: Occupational status, educational status, Marital status, Monthly income, Household wealth, Oslo social support status, perceived social position, presence of alcohol drinking problem, Presence of Khat chewing related problem, age at marriage, Religion, Perceived economic position.

*** Variable(s) entered on step 1: Occupational status, educational status, Marital status, number of children (biological), Monthly income, Household wealth, Oslo social support status, perceived economic position, perceived social position, age group.

Table 7. Current reproductive health, Social, and economic related correlates of common mental health problem of women who were in Woldia during the wartime, Woldia, north Ethiopia, April 2023

Characteristics	Depression [PHQ-9 score ≥ 10]		Generalized Anxiety Disorder (GAD) [Moderate or Sever GAD]		Post-traumatic stress disorder [PTSD]	
	Crude OR (95% CI)	Adjusted* OR (95% CI)	Crude OR (95% CI)	Adjusted** OR (95% CI)	Crude OR (95% CI)	Adjusted*** OR (95% CI)
Age at marriage						
< 15 yrs	1.00	= =	1.00	1.00	1.00	= =
15-17 yrs	1.56 (0.95, 2.56)	= = = = = = = = = =	1.46 (0.87, 2.47)	1.44 (0.81, 2.57)	0.93 (0.58, 1.50)	= = = = = = = = = =
18 yrs. or more.	1.45 (0.92, 2.29)	= = = = = = = = = =	1.69 (1.04, 2.75)	1.70 (0.98, 2.94)	0.94 (0.61, 1.45)	= = = = = = = = = =
Number of children						
No child	1.00	= =	1.00	= =	1.00	1.00
1-2 children	0.88 (0.59, 1.30)	= = = = = = = = = =	0.92 (0.61, 1.38)	= = = = = = = = = =	0.59 (0.40, 0.87)	0.89 (0.54, 1.48)
3 or more	0.86 (0.57, 1.29)	= = = = = = = = = =	0.90 (0.59, 1.37)	= = = = = = = = = =	0.54 (0.36, 0.81)	0.67 (0.39, 1.17)
Wealth index						
Lower wealth	1.00	1.00	1.00	1.00	1.00	1.00
Medium wealth	0.38 (0.29, 0.48)	0.45 (0.34, 0.60)	0.38 (0.29, 0.48)	0.40 (0.30, 0.54)	0.49 (0.39, 0.83)	0.58 (0.43, 0.78)
Higher wealth	0.21 (0.15, 0.29)	0.33 (0.21, 0.53)	0.20 (0.15, 0.28)	0.28 (0.17, 0.46)	0.62 (0.49, 0.77)	0.45 (0.28, 0.72)
Perceived economic status.						
Higher	0.28 (0.16, 0.50)	0.94 (0.37, 2.40)	0.27 (0.14, 0.50)	0.74 (0.26, 2.14)	0.49 (0.29, 2.33)	= = = = = = = = = =
Moderate	0.56 (0.46, 0.72	1.17 (0.82, 1.67)	0.64 (0.51, 0.80)	1.27 (0.88, 1.84)	0.74 (0.50, 1.08)	= = = = = = = = = =
Lower	1.00	1.00	1.00	1.00	1.00	= =
Perceived social status.						
Higher	0.29 (0.19, 0.47)	0.70 (0.34, 1.44)	0.33 (0.20, 0.53)	0.89 (0.42, 1.92)	0.47 (0.30, 0.72)	1.00 (0.51, 1.97)
Moderate	0.48 (0.38, 0.59)	0.72 (0.52, 0.99)	0.56 (0.45, 0.70)	0.88 (0.63, 1.23)	0.44 (0.35, 0.55)	0.51 (0.36, 0.71)
Lower	1.00	1.00	1.00	1.00	1.00	1.00
Oslo-social support index						
Poor	1.00	1.00	1.00	1.00	1.00	1.00
Moderate	0.52 (0.41, 0.65)	0.71 (0.54, 0.95)	0.61 (0.48, 0.77)	0.69 (0.53, 0.90)	0.88 (0.70, 1.11)	1.35 (1.01, 1.80)
Strong	0.24 (0.16, 0.48)	0.27 (0.17, 0.49)	0.28 (0.19, 0.41)	0.34 (0.22, 0.52)	0.42 (0.30, 0.61)	0.53 (0.33, 0.85)
Spousal alcohol-related prob.						
No	1.00	1.00	1.00	1.00	1.00	= =
Yes	1.74 (1.26, 2.41)	1.42 (0.94, 2.16)	1.61 (1.16, 2.24)	1.36 (0.0.89, 2.07)	1.14 (0.81, 1.59)	= = = = = = = = = =
Spousal Khat-related prob.						
No	1.00	1.00	1.00	1.00	1.00	= =
Yes	1.92 (1.15, 3.21)	2.41 (1.25, 4.65)	1.75 (1.05, 2.94)	1.84 (0.94, 3.57)	1.38 (0.82, 2.33)	= = = = = = = = = =

* Depression: Age grouped, Occupational status, educational status, Marital status, Monthly income, Household wealth, Oslo social support status, perceived economic position, perceived social position, presence of alcohol drinking problem, Presence of Khat chewing-related problem.

** Variable(s) entered on step 1: Occupational status, educational status, Marital status, Monthly income, Household wealth, Oslo social support status, perceived social position, presence of alcohol drinking problem, Presence of Khat chewing related problem, age at marriage, Religion, Perceived economic position.

*** Variable(s) entered on step 1: Occupational status, educational status, Marital status, number of children (biological), Monthly income, Household wealth, Oslo social support status, perceived economic position, perceived social position, age group.

Discussion

In Woldia, one year after wartime, the study found alarming rates of common mental health problems among women. Sixty-one percent felt life was a miserable consequence, 32.2% showed to have moderate to severe depression, while 5% had severe depression. Similarly, 22.7% had moderate generalized anxiety disorder (GAD), 10.5% had severe GAD, and 44.3% exhibited symptoms of post-traumatic stress disorder (PTSD). Almost half of the women had major symptoms of either of the three common mental health problems, of which about 33% showed having comorbidity of two or more of these disorders. Various risk and protective factors were identified: depressive symptoms were more prevalent among older, single women and those with spouses using khat, while women with higher wealth indices and strong social support were less likely to suffer. Similar patterns were observed for GAD and PTSD. Older age, single status, and moderate-income were risk factors, while a higher wealth index and stronger social support offered some protection.

The findings from Woldia one year after wartime present a disturbing picture of the mental health conditions among women in the area, with elevated rates of depression, generalized anxiety disorder (GAD), and post-traumatic stress disorder (PTSD). The magnitude of common mental illness is too huge to compare with people living in stable conditions. Compared to global averages, these rates are exceedingly high. For instance, the World Health Organization estimates that, globally, around 4.4% of the population suffers from depression [27, 28] and 3.6% from anxiety disorders (28). In contrast, our study in Woldia found that 32.2% of women showed signs of moderate to severe depression, and 22.7% had moderate GAD. Such drastic differences underscore the devastating mental health impact of wartime experiences on women in developing countries like Woldia, where post-war poverty is high, and access to mental health services is often limited.

Furthermore, the prevalence of PTSD among women in Woldia, at 44.3%, also stands out as alarming when compared to other conflict and post-conflict regions. Research in other developing countries affected by

conflict, such as Rwanda and Bosnia, has reported PTSD rates ranging from 20% to 40% [29], suggesting that the situation in Woldia is at the higher end of the distress scale. The high percentage of women who feel their life in misery stresses attending life in poverty and lacking a meaningful life (61%) is another concerning metric that points to existential crises, often less quantified but deeply impactful in terms of long-term well-being. The findings from Woldia, reflecting the mental health conditions of women in the aftermath of wartime, are deeply concerning. Almost half of the women exhibited significant symptoms associated with one of the three common mental health problems. This high prevalence rate highlights the profound psychological trauma that such conflicts can inflict on vulnerable populations, especially women in developing regions. Comparatively, in many global contexts outside of conflict zones, the prevalence of such mental health disorders is typically much lower. The added distress is attributed to the direct and indirect impacts of the war, including loss of loved ones, physical harm, displacement, and overarching social disruption.

Moreover, approximately 33% of these women demonstrated comorbidity – or the co-occurrence of two or more disorders – which underscores the complexity and depth of their mental health challenges. Comorbidity often suggests that affected individuals experience more severe psychological distress, significantly complicating treatment pathways. For instance, treating a person with depression alone differs from treating someone with depression and generalized anxiety disorder. The combined impact of comorbid disorders can lead to exacerbated symptoms, prolonged suffering, and increased disability. In other global contexts, particularly in developed nations, there is an established recognition of the challenges posed by comorbidity, with specific treatment regimens designed to address such complexities.

Given the grim picture painted by these findings, it becomes imperative to consider the broader implications for Woldia and similar regions. Women are foundational pillars in the fabric of society, often holding familial

and community structures together. If their mental well-being is compromised, the ripple effect can impact families, children's development, community cohesion, and societal progress.

The relationship between socio-demographic factors and mental health outcomes among women in Woldia mirrors findings from other studies, affirming the global relevance of these connections. Previous research has demonstrated a higher prevalence of depressive symptoms among older individuals [30] and single women [31]. Similarly, spouse substance use, such as khat chewing, has been identified as a stressor contributing to mental health conditions in other contexts [32]. These risk factors apply consistently to generalized anxiety disorder (GAD) and post-traumatic stress disorder (PTSD), suggesting that age, marital status, and spousal behaviors are critical determinants across various mental health disorders.

The study also sheds light on protective factors, namely higher wealth indices and strong social support, associated with lower rates of depressive symptoms, GAD, and PTSD. These findings align with a body of literature emphasizing the importance of social capital and economic stability in mental health outcomes [33]. Social support, often measured through tools like the Oslo-social support index, has been shown to provide a buffer against the adverse psychological impacts of stress and trauma [34] and is particularly important in the context of developing nations where formal mental health services may be lacking.

The consistent association of risk and protective factors across the three major mental health issues of depression, GAD, and PTSD suggests that interventions to improve mental health in this context should adopt a multi-faceted approach. Addressing root causes like substance abuse in spouses and providing targeted mental health services for older, single women could be critical steps. Simultaneously, community-based programs that enhance social support networks and economic empowerment initiatives aimed at improving the wealth index of women can serve as effective preventative measures [35]. Given the multi-dimensional nature

of these risk and protective factors, interdisciplinary strategies involving social, economic, and healthcare sectors may offer the most effective pathway to mitigate these pressing mental health concerns.

The study on mental health issues among women in Woldia after wartime provides crucial insights. Still, it has limitations in terms of generalizability, temporal scope, and depth of analysis of protective factors. Specifically, the focus on a region deeply affected by conflict and post-war conditions may limit the study's applicability to other areas. The cross-sectional nature of the research provides a "snapshot" rather than a longitudinal view, potentially missing evolving trends in mental health. Lastly, while protective factors like wealth indices and social support are mentioned, a more in-depth exploration of these elements, such as the types and quality of social support and their longitudinal impact, could offer a more nuanced understanding of their role in mental health outcomes.

Conclusion

The findings from Woldia reveal a severe mental health crisis among women in the aftermath of wartime, with elevated levels of depression, anxiety, and PTSD far exceeding global averages. This situation not only jeopardizes the well-being of women but also has far-reaching implications for families and communities. Addressing this crisis is urgent and requires a multi-pronged approach, considering both risk and protective factors identified in the study.

Recommendations

Given the dire situation, immediate and sustained interventions are essential. Multi-disciplinary teams should be deployed to provide targeted mental health services, particularly for vulnerable groups like older and single women. Community-based initiatives should be implemented to strengthen social support networks and offer economic empowerment. Coordination between social, financial, and healthcare sectors is imperative for

creating a holistic support system that can address the complex challenges posed by high rates of mental disorders and comorbidities among women in Woldia and similar conflict-affected areas.

Authors' contributions

a. Contributed to conceptualizing, writing, data collection tools, analysis, rewriting, and reviewing

b. Contributed to data collection tools, analysis, and review and editing

c. Contributed to conceptualizing, data collection tools, analysis, rewriting, and reviewing

Acknowledgments

We want to acknowledge the contributions of the following groups: the Woreda Health Office and the Woreda Women's and Children's Affairs Office of Woldia town for their positive consent and support in using district data for sampling. We extend our gratitude to the enumerators who collected the data in the field and the supervisors who diligently assisted and monitored the data collectors. Finally, we are deeply grateful to the women who participated and consented to share their real experiences.

Supporting Information

S1 Text. Ethical approval for Prof Negussie.

Reference

1. Kar N, Bastia BK. Post-traumatic stress disorder, depression and generalised anxiety disorder in adolescents after a natural disaster: a study of comorbidity. Clinical Practice and Epidemiology in Mental Health. 2006;2(1):1-7.

2. Naqvi H, Khan MM. Depression in primary care: difficulties and paradoxes. Journal of Pakistan Medical Association. 2005;55(9):393.

3. Marques L, Robinaugh DJ, LeBlanc NJ, Hinton D. Cross-cultural variations in the prevalence and presentation of anxiety disorders. Expert Review of Neurotherapeutics. 2011;11(2):313-22.

4. Perkonigg A, Kessler RC, Storz S, Wittchen HU. Traumatic events and post-traumatic stress disorder in the community: prevalence, risk factors and comorbidity. Acta psychiatrica scandinavica. 2000;101(1):46-59.

5. Kar N, Bastia BK. Post-traumatic stress disorder, depression and generalised anxiety disorder in adolescents after a natural disaster: a study of comorbidity. Clinical Practice and Epidemiology in Mental Health. 2006;2(1):17. doi: 10.1186/1745-0179-2-17.

6. Reddy MS. Depression: the disorder and the burden. Indian journal of psychological medicine. 2010;32(1):1-2. Epub 2010/01/01. doi: 10.4103/0253-7176.70510. PubMed PMID: 21799550; PubMed Central PMCID: PMCPMC3137804.

7. Hoppen TH, Priebe S, Vetter I, Morina N. Global burden of post-traumatic stress disorder and major depression in countries affected by war between 1989 and 2019: a systematic review and meta-analysis. BMJ global health. 2021;6(7):e006303.

8. Knifton L, Inglis G. Poverty and mental health: policy, practice and research implications. BJPsych bulletin. 2020;44(5):193-6. Epub 2020/08/04. doi: 10.1192/bjb.2020.78. PubMed PMID: 32744210; PubMed Central PMCID: PMCPMC7525587.

9. Fortuna LR, Porche MV, Alegria M. Political violence, psychosocial trauma, and the context of mental health services use among immigrant Latinos in the United States. Ethnicity & health. 2008;13(5):435-63. Epub 2008/10/14. doi: 10.1080/13557850701837286. PubMed PMID: 18850369; PubMed Central PMCID: PMCPMC2771411.

10. Yimer S, Girma Z, Mengistu N, Shumye S. Determinants of Common Mental Disorders Among People Living with HIV/AIDS Attending ART Service in Southern Ethiopia. HIV/AIDS-Research and Palliative Care. 2021:917-25.

11. Knifton L. Understanding and addressing the stigma of mental illness with ethnic minority communities. Health Sociology Review. 2012;21(3):287-98. doi: 10.5172/hesr.2012.21.3.287.

12. Hietamäki J, Huttunen M, Husso M. Gender Differences in Witnessing and the Prevalence of Intimate Partner Violence from the Perspective of Children in Finland. International journal of environmental research and public health. 2021;18(9). Epub 2021/05/01. doi: 10.3390/ijerph18094724. PubMed PMID: 33925260; PubMed Central PMCID: PMCPMC8125222.

13. Dubé C, Gagné MH, Clément M, Chamberland C. Community Violence and Associated Psychological Problems Among Adolescents in the General Population. Journal of child & adolescent trauma. 2018;11(4):411-20. Epub 2018/12/14. doi: 10.1007/s40653-018-0218-8. PubMed PMID: 30546818; PubMed Central PMCID: PMCPMC6267123.

14. Solomon P, Draine J. Examination of grief among family members of individuals with serious and persistent mental illness. Psychiatric Quarterly. 1996;67(3):221-34. doi: 10.1007/BF02238953.

15. Makango B, Alemu ZA, Solomon T, Lemma N, Girma T, Mohammednur T, et al. Prevalence and factors associated with post-traumatic stress disorder among internally displaced people in camps at Debre Berhan, Amhara Region, Ethiopia: a cross-sectional study. BMC Psychiatry. 2023;23(1):81. doi: 10.1186/s12888-023-04570-w.

16. Kwon M, Kim H. Psychological Well-Being of Female-Headed Households Based on Age Stratification: A Nationwide Cross-Sectional Study in South Korea. International journal of environmental research and public health. 2020;17(18). Epub 2020/09/10. doi: 10.3390/ijerph17186445. PubMed PMID: 32899644; PubMed Central PMCID: PMCPMC7559352.

17. Chandra PS, Herrman H, Fisher J, Kastrup M, Niaz U, Rondón MB, et al. Contemporary Topics in Women's Mental Health. doi: 10.1002/9780470746738.

18. Worku A, Jemal M, Gedefaw A. Institutional delivery service utilization in Woldia, Ethiopia. Science journal of public health. 2013;1(1):18-23. doi: 10.11648/j.sjph.20130101.13.

19. Central Statistics Authority Ethiopia. Third National Population and Housing Census in May and November 2007. Addis Ababa2010.

20. Deyessa N, Berhane Y, Alem A, Ellsberg M, Emmelin M, Hogberg U, et al. Intimate partner violence and depression among women in rural Ethiopia: a cross-sectional study. Clinical practice and epidemiology in mental health. 2009;5(1):1-10.

21. Kroenke K, Spitzer RL, Williams JB. The PHQ-9: validity of a brief depression severity measure. Journal of general internal medicine. 2001;16(9):606-13.

22. Manzar MD, Salahuddin M, Alghadir A, Anwer S, Peter S, Bahammam AS, et al. Psychometric properties of the Generalized Anxiety Disorder-7 Scale in Ethiopian university students. Bulletin of the Menninger Clinic. 2021;85(4):405-27.

23. Spitzer RL, Kroenke K, Williams JB, Löwe B. A brief measure for assessing generalized anxiety disorder: the GAD-7. Archives of internal medicine. 2006;166(10):1092-7.

24. Bovin MJ, Marx BP, Weathers FW, Gallagher MW, Rodriguez P, Schnurr PP, et al. Psychometric properties of the PTSD checklist for diagnostic and statistical manual of mental disorders–fifth edition (PCL-5) in veterans. Psychological assessment. 2016;28(11):1379.

25. Garland AF, Deyessa N, Desta M, Alem A, Zerihun T, Hall KG, et al. Use of the WHO's Perceived Well-Being Index (WHO-5) as an efficient and potentially valid screen for depression in a low income country. Families, Systems, & Health. 2018;36(2):148.

26. World Health Organization. Researching violence against women: practical guidelines for researchers and activists, accessed on June 24, 2024, https://www.who.int/publications/i/item/9241546476: World Health Organization; 2005.

27. World Health Organization L, Organization WH. Global and regional estimates of prevalence: Depressive disorders. Depression and other common mental disorders: Global health estimates. 2017:8-9. doi: http://www.jstor.org/stable/resrep28026.6.

28. Organization WH. Number of people with depression increases. World Health Organization, Geneva. 2017.

29. Ng LC, Stevenson A, Kalapurakkel SS, Hanlon C, Seedat S, Harerimana B, et al. National and regional prevalence of posttraumatic stress disorder in sub-Saharan Africa: A systematic review and meta-analysis. PLoS medicine. 2020;17(5):e1003090. Epub 2020/05/16. doi: 10.1371/journal.pmed.1003090. PubMed PMID: 32413027; PubMed Central PMCID: PMCPMC7228043.

30. Kasa AS, Lee S-C, Chang H-C. Prevalence and factors associated with depression among older adults in the case of a low-income country, Ethiopia: a systematic review and meta-analysis. BMC Psychiatry. 2022;22(1):675. doi: 10.1186/s12888-022-04282-7.

31. Davies L, Avison WR, McAlpine DD. Significant life experiences and depression among single and married mothers. Journal of Marriage and the Family. 1997:294-308. doi: org/10.2307/353471.

32. Lander L, Howsare J, Byrne M. The impact of substance use disorders on families and children: from theory to practice. Social work in public health. 2013;28(3-4):194-205. Epub 2013/06/05. doi: 10.1080/19371918.2013.759005. PubMed PMID: 23731414; PubMed Central PMCID: PMCPMC3725219.

33. De Silva MJ, Huttly SR, Harpham T, Kenward MG. Social capital and mental health: a comparative analysis of four low income countries. Social science & medicine. 2007;64(1):5-20. doi: https://doi.org/10.1016/j.socscimed.2006.08.044.

34. Thoits PA. Mechanisms linking social ties and support to physical and mental health. Journal of health and social behavior. 2011;52(2):145-61. Epub 2011/06/16. doi: 10.1177/0022146510395592. PubMed PMID: 21673143.

35. Patel V, Saxena S, Lund C, Thornicroft G, Baingana F, Bolton P, et al. The Lancet Commission on global mental health and sustainable development. The lancet. 2018;392(10157):1553-98.

Click here to access/download
Supporting Information
S1 text
Ethical approval for Prof. Negussie.pdf

Click here to access/download

Supporting Information - Compressed/ZIP File Archive

S2 supportive data collected and zipped in spss.zip
