# Supplementary material for: The impact of wartime conflict on the mental health problems of women in the conflict-hit population in Woldia, Ethiopia
Source: PLoS One. 2025 Jun 9;20(6):e0322047. doi: 10.1371/journal.pone.0322047 (PMC12148197; doi:10.1371/journal.pone.0322047)
Supplement: Attachment — Submitted filename: Review of Manuscript_PlosOne_AJ.pdf [file pone.0322047.s003.pdf]

Review of Manuscript

The impact of wartime conflict on mental health problems of women in conflict-hit population in Woldia, Ethiopia

PONE-D-24-23457

This is an important and valuable study that addresses critical mental health issues in post-conflict settings. Evidence from such regions is vital as it highlights specific needs and provides actionable recommendations. The study is particularly noteworthy given the challenges associated with recruiting over 1,500 women in an area still experiencing severe security concerns. The large sample size (n = 1505) strengthens the reliability and generalizability of the findings.

Strengths:

- **Significance of the Study:** The study contributes crucial evidence on mental health in post-conflict settings, where data is often scarce.
- **Large Sample Size:** Including over 1,500 women is a significant achievement, particularly in such a challenging and resource-limited context.
- **Well-Summarized Findings:** The sections summarizing the mental health impact of conflict (e.g., Lines 71–79) are clear and concise.

Minor Corrections and Suggestions:

1. **Line 57:**
    - **Original:** "can be all too familiar"
    - **Suggestion:** Rephrase as "can be all too common" for clarity.
2. **Line 61:**
    - **Clarification:** "Depression, the typical psychological condition"
    - **Suggestion:** Replace “typical” with “most common” to avoid ambiguity.
3. **Line 80:**
    - **Improvement:** Add a better transition to the next paragraph. Suggested phrasing:
      “On top of these mental health stressors in developing countries, wartime has a severe negative impact on mental health, particularly for women.”
4. **Line 98 Onwards (Impact on Child Rearing):**
    - Consider elaborating on the impact of maternal mental disorders on children. For instance:
      “Depression or PTSD may hinder a mother’s ability to interact with her child, potentially leading to neglect or even violent behavior, which can perpetuate trauma transgenerationally.”
5. **Line 111:**
    - Add: *“This may also support recommendations for targeted actions.”*
6. **Addiction and Violence Cycles:**
    - Highlight the importance of scaling up addiction treatment services (regarding spouses) in the region to break the cycle of violence and substance abuse.
7. **Line 196 & 202:**
    - Check for consistent use of past tense throughout the manuscript.
8. **Table 1:**

- While acceptable, the table could benefit from improved formatting for readability. This may, however, be addressed during the journal's editing process.

9. **Statistical Analysis:**
   - Consider including a correction for multiple testing if applicable.
10. **Line 339:**
    - **Original:** "The magnitude of common mental illness is too huge to compare with people living in stable conditions."
    - **Suggestion:** Rephrase for clarity, e.g.: *"The burden of common mental illnesses in post-conflict areas is significantly higher compared to populations in stable settings."*
11. **Line 341 (Prevalence Data):**
    - Provide 1-year prevalence rates of depression for comparison. For example, the 1-year prevalence of depression is approximately 15%.
12. **Line 343 (Local Prevalence):**
    - Include local pre-conflict prevalence data for Ethiopia, particularly Northern Ethiopia, and compare it with your prevalences. Reference other relevant studies or reports on post-conflict mental health in the region, including war trauma and GBV statistics.
    - Are there publications describing the war-trauma experienced in the region?
13. **Line 389 (Trauma-Informed Care):**
    - Given the high prevalence of GBV and PTSD symptoms, recommend trauma-informed care and psychotherapy, including community-based approaches.
    - Suggest training healthcare workers and laypeople to provide trauma-sensitive support.
14. **Lines 387 and 408:**
    - Add survivors of GBV to the discussion of vulnerable groups, alongside older and single women.
15. **Funding:** You indicate that no funding was received. 18 data collectors had been employed for the study. How was that funded or did all oft hem contribute volunteering? Please indicate int he acknowledgements.

Conclusion:

This study makes a significant contribution to understanding the mental health burden in the post-conflict setting of Northern Ethiopia, with a strong focus on women. Addressing the minor revisions noted above will further strengthen the manuscript and ensure its findings have the maximum impact. The recommendations, particularly for trauma-informed care and addiction treatment, are essential steps in addressing the ongoing mental health crisis in the region.
