# Supplementary material for: The impact of wartime conflict on the mental health problems of women in the conflict-hit population in Woldia, Ethiopia
Source: PLoS One. 2025 Jun 9;20(6):e0322047. doi: 10.1371/journal.pone.0322047 (PMC12148197; doi:10.1371/journal.pone.0322047)
Supplement: Attachment — Submitted filename: Response to reviewers_Mar 01 2025.docx [file pone.0322047.s004.docx]

**To: PLOS ONE journal Editor-in-chief and Reviewers (manuscript ID:** PONE-D-24-23457**)**

**Dear Editor and reviewers**,

On behalf of our author group, we sincerely thank the Editor-in-Chief and the reviewers for their insightful and constructive feedback on our manuscript, “The Impact of Wartime Conflict on Mental Health Problems of Women in Conflict-Hit Populations in Woldia, Ethiopia.” The reviewers’ thoughtful comments and valuable suggestions have significantly enhanced the quality and clarity of our manuscript. We have carefully considered each point raised and have provided detailed responses, outlining how we addressed the concerns and tracking all revisions made accordingly. We truly appreciate the time and effort dedicated to reviewing our work.

In the attached file, we have structured our responses: the first column presents the reviewers’ comments, the second column contains our responses typically acknowledging and incorporating the suggested improvements, and the third column highlights the specific changes made in the manuscript, with modifications marked. We hope this format facilitates the review process. Once again, we sincerely thank you for your invaluable feedback and guidance.

Sincerely,

# Response to the Reviewers of Manuscript

**Journal Requirements:**

| Comments | Response to comments | Correction page & line |
| --- | --- | --- |
| 1. When submitting your revision, we need you to address these additional requirements. | We will try to adjust and follow the requirements of the journal style. | NA |
| 2. Please provide additional details regarding participant consent. In the ethics statement in the Methods and online submission information, please ensure that you have specified (a) whether consent was informed and (b) what type you obtained (for instance, written or verbal, and if verbal, how it was documented and witnessed). If your study included minors, state whether you obtained consent from parents or guardians. If the need for consent was waived by the ethics committee, please include this information. | We have used ethical approval from the Ethiopian Public Health Association (IRB) after experts reviewed the issue. Now it has been updated in detail, what has been done, during the interview.  Consent was obtained from 1) community (village) leaders and individuals included in the study. 2) Since the study included a higher number of uneducated adult mothers, we obtained it orally, and the witness was only by the enumerator, as it should not include a third party]. Enumerators were given training on following the WHO ethics for VAW. 3) All participants were adults 18 or above, not needing consent from a guardian. | Line 221-237 |
| 3. We are unable to open your Supporting Information file “data, Mar 08.sav”. Please kindly revise as necessary and re-upload. | Sorry, but I will re-send it and put it on a repository to access it. | N/A |
| 4. Please review your reference list to ensure that it is complete and correct. If you have cited papers that have been retracted, please include the rationale for doing so in the manuscript text, or remove these references and replace them with relevant current references. Any changes to the reference list should be mentioned in the rebuttal letter that accompanies your revised manuscript. If you need to cite a retracted article, indicate the article’s retracted status in the References list and also include a citation and full reference for the retraction notice. | We are using endnote 21, and we put the reference as they support the issue we raised as a testimony. We will consider all the possibilities for references. | N/A |
| **Reviewer #1:** |  |  |
| **Reviewer #1:** • Although the abstract is well-structured and informative, it could benefit from improvement to enhance clarity and precision. Replacing vague terms like “major symptoms” and “multi-faceted approach” with more specific language would enhance the presentation of findings and recommendations. Additionally, refining “post-wartime” to “post-conflict” would align with more standard terminology. The methods section could also benefit from a brief mention of participant inclusion and exclusion criteria, as well as a clearer explanation of the systematic sampling process. These adjustments would strengthen the abstract’s overall clarity and impact. | Thank you for the comments, and we have tried our best to replace the vague terms you mentioned.  The methods session of the abstract briefly described the inclusion/ exclusion criteria related to the number of words in the abstract [we will try our best to show it]. Still, it is well illustrated in the methods session of the body of the manuscript. | Line 38, 418  Line 125-128 |
| • The introduction lays a solid foundation but could benefit from clearer focus and refinement. The detailed descriptions of GAD, PTSD, and depression (lines 47–58) could be shortened to emphasize their relevance in the context of wartime. Additionally, the discussion of the global burden of mental health disorders (lines 59–70) could more strongly highlight the importance of addressing these issues in developing countries like Ethiopia, particularly in the context of the North Ethiopian conflict. The section on infectious diseases (lines 75–76) seems unrelated to the study’s objectives and could be removed unless it directly supports the focus of the research. | We are happy with your comments, and focusing the introduction on the issue we want to describe is crucial. It was planned to show the problem (common mental disorders), in general, is public health important, having tried to explain its common contributing factors, and it is later focused on common mental health as related to wartime conflicts. Now, we have amended it.  I have shortened lines 47-58, including 59-70, and we have emphasized the wartime conflicts related to common mental health. We have also taken out the issue of infectious disease as related to mental illness. | Re-reading the whole part of the introduction. Line shortened so not visible,  Line 53-57 is omitted, and put at line 54  Line 61-70 is omitted, now at line 58 |
| • While lines 80–102 offer valuable insight into the impacts of war on women’s mental health, there is some repetition, particularly regarding trauma and its effects. For instance, the mention of PTSD resulting from trauma (lines 56–57) and repeated references to stress caused by war (lines 90–93) could be combined to avoid redundancy. The introduction begins to focus on the North Ethiopian context only in lines 103–109. Shifting this earlier in the introduction would help establish the study’s specific focus more quickly and create a stronger connection between the global and local perspectives. | Yes, as I see it, the above consideration is given too. But the first part was related to making people understand the common mental health problems and what they are, and as an important issue [showing its severity and distribution in developing countries]. It is true to go directly to the issue, but the other reviewer wanted to expand it but not repeat issues. Lines 56-57 and 90-93 seem not similar, so no measure was taken. | Lines as in the above. |
| Methods |  |  |
| • The methods section provides a thorough overview, but there are areas that can be improved for clarity and precision. In lines 115–123, while the setting is adequately described, there could be a clearer focus on how Woldia’s context specifically impacts the study’s objectives. The section on inclusion and exclusion criteria (lines 124–130) can be streamlined for brevity. For example, rather than listing each exclusion category separately, these could be grouped or summarized more succinctly. In lines 131–145, the sampling strategy is comprehensive, but the connection to the research aims could be made more explicit to ensure the sampling process is fully aligned with the study’s objectives. | Thank you for the comment on the setting. It has been amended to include the war-related situation in Woldia.  For the inclusion-exclusion, we have tried to bring it into one, reducing the repeatability.  Compared with the objective, since the study is part of assessing IPV, caution was given to follow the ethical issues of keeping privacy, and some people who were not able to consent or had difficulty communicating were excluded. | Line 116-122  Line 221-237 |
| • In the data analysis section (lines 201–219), the use of logistic regression and multivariable analysis is appropriate. However, lines 199–200 introduce the concept of effect-modifying variables, but the relationship between these variables and the study outcomes should be clarified further. This will enhance understanding of how these variables may influence the results. The ethical considerations (lines 220–225) mention informed consent, but elaborating on how participant confidentiality was maintained during data collection would strengthen this section. Additionally, tightening some sentences for conciseness and improving transitions between sections would improve the overall readability and flow. | In this manuscript, we did not assess the variables list’s mediator, effect modifier, or interaction effect. At the initial stage of the proposal, we planned to determine mediation and effect modification. Still, in this study, we didn’t do it, as it needs special analysis and its objective modified. Therefore, we have modified the analysis session and included what we did in this manuscript. | Line 189-194  And  Line:  The last paragraph of the analysis session |
| Results |  |  |
| • In line 230, the sentence “A prominent segment, accounting for 41.7%, fell into the age group between 25 to 34 years, with a mean age of 36.2 + 10.2, spanning an age range of 18 to 60 years” could be broken into simpler sentences to improve readability. A clearer alternative could be: “The majority of participants (41.7%) were aged between 25 and 34 years. The mean age of participants was 36.2 years, with an age range of 18 to 60 years.” This will improve sentence flow and make the information easier to digest. Similar improvements can be made in other areas where multiple statistics are stacked in one sentence, such as line 241: “The study revealed that over 37% of participants reported a monthly income below 1,500 Ethiopian Birr, with two-thirds earning less than 3,000 Ethiopian Birr (equivalent to $60).” This could be split for clarity: “Over 37% of participants reported a monthly income below 1,500 Ethiopian Birr. Two-thirds earned less than 3,000 Ethiopian Birr (approximately $60).” | Thank you very much for the comment. It is well accepted and modified accordingly! | Line 239 241  Lines 250 and 255 |
| • Ensure consistency in the formatting of percentages. In lines 236–237, for instance, the phrase “approximating ten percent” can be made more precise, e.g., “approximately 10%” or “10%”. This ensures consistency in numerical representation across the section. Additionally, check for consistency in the presentation of data in tables and narratives (lines 235–236). Some data points, like “87.2%” and “10.7%” (line 234), could be followed by additional explanations to clarify their importance or relation to other figures. | It is true to be consistent in writing numbers vs. alphabetical writing-ups. When we approximate, we may not need the decimals, while if we use the table, we should write the exact number with decimals. Thus, it is well amended. Thank you again. | Line 243 and 245  Correction goes to all the results sessions. |
| • Transitioning smoothly from one section to another will enhance the coherence of the text. For example, in line 250, you move into economic challenges attributed to spouses’ alcohol and khat consumption. A brief sentence linking these findings to the previous socioeconomic section would help. For instance: “In addition to income and social status, other familial factors also played a significant role in the participants’ economic well-being, with some facing challenges due to spouses’ substance use.” | It is well-accepted that we have directly used your connecting sentences. Thank you for your comments and supplementation. | Line 258-260 |
| • The section on mental health (lines 256–273) could be more effectively organized by grouping similar findings together. For example, present all the mental health disorders in one subsection and break them into categories (e.g., depression, anxiety, PTSD) to improve clarity. Lines 257–266 could benefit from such organization: first, describe the general rates of depression, anxiety, and PTSD, then break them down further (e.g., percentage of participants with severe symptoms). | We want to thank you for those comments. We believed in showing first general and then specifically each component of the mental health problems. | Line 267 to 274 |
| • The use of terms like “Oslo-social support index” (lines 248–249) should be briefly explained or defined, particularly for readers unfamiliar with these metrics. You could add a short explanation, such as: “The Oslo-social support index, which measures perceived social support, was used to assess the social well-being of participants.” | This is also a strong comment that should be well-defined. We have made a detailed description of the measurements in the methods session, and we would like to thank you again. | Line 189-to-194 |
| Discussion |  |  |
| • The discussion provides important insights into the mental health challenges faced by women in Woldia after the conflict, but some areas can be refined to improve clarity and depth. First, the comparison with global averages (lines 340–344) is valuable, yet the argument could be strengthened by further contextualizing the impact of war-related stressors such as displacement, loss, and social disruption on mental health (lines 337–338, 345). Exploring how these stressors interact with individual characteristics like age, marital status, and socioeconomic factors (lines 333–335, 373–377) would offer a deeper understanding. Additionally, while the section on comorbidity (lines 359–363) is crucial, it would benefit from an expanded discussion on treatment strategies or interventions specifically designed for women with multiple mental health conditions, particularly in settings with limited resources (lines 365–366). | We have amended the discussion session as this part needs expansion and interpreting from different perspectives, and we want to thank you for the positive response. | Line 362-366  Line 398-405  Line 409-415  Line 421-430 |
| • The discussion of socio-demographic risk and protective factors (lines 371–380) aligns well with existing literature, but it would be helpful to delve into how these factors might present differently in conflict-affected areas (lines 373–376). For instance, exploring why certain protective factors, such as social support (lines 379–383), are more effective in post-conflict situations would provide additional insights. The suggestion of a multi-faceted intervention approach (lines 384–391) is commendable, but it would be even more valuable to describe how such strategies could be practically implemented in Woldia, offering a clear pathway for policymakers and practitioners. Lastly, while the study acknowledges its limitations (lines 392–399), it would be useful to suggest specific ways future research could address these gaps, such as conducting longitudinal studies to track changes in mental health over time (lines 395–396) and exploring the quality of social support networks (lines 397–398). | Since we got similar comments from the other reviewer, thank you both. We have tried to amend the use of local context in the region.  Amendments related to exploring the reasons for the risk and protective factors are made at each level and in common.  What to do to mitigate the risk factors and increase the existence of the protective factors has been added, although it was good to do so in the recommendation section. | Line 362-366  Line 409-415  Line 421-430 |
| Conclusion and recommendation |  |  |
| • The conclusion and recommendations section effectively summarizes the findings and suggests practical steps to address the mental health crisis in Woldia. However, the conclusion could be enhanced by incorporating more specific details from the study’s findings, such as the prevalence of comorbidity (lines 359–363) and its implications for treatment (lines 365–366), offering a more comprehensive understanding of the mental health challenges faced by women in the region. Furthermore, the recommendations could be more detailed by providing concrete actions or examples of successful interventions in similar contexts. This could include practical strategies for mobilizing multi-disciplinary teams and examples of effective community-based programs in other post-conflict areas. Such additions would strengthen the recommendations and provide actionable guidance for stakeholders addressing the mental health crisis in Woldia. | Thank you for your comments. However, conclusions and recommendations should generally be kept broad and based on the research findings. While detailed explanations may be necessary in the discussion section to explore possible pathways and mechanisms, the conclusion should focus on summarizing the findings and providing relevant recommendations. Therefore, our group has limited the conclusion to the key findings and corresponding recommendations but is happy with the comments. | NA |

| Comments | Response to comments | Correction page & line |
| --- | --- | --- |
| This is an important and valuable study that addresses critical mental health issues in post-conflict settings. Evidence from such regions is vital as it highlights specific needs and provides actionable recommendations. The study is particularly noteworthy given the challenges of recruiting over 1,500 women in an area still experiencing severe security concerns. The large sample size (n = 1505) strengthens the reliability and generalizability of the findings. | We appreciate the reviewer for providing us with the overall positive manuscript we have, and we want to thank the reviewers group. | NA |
| - **Significance of the Study:** The study contributes crucial evidence on mental health in post-conflict settings, where data is often scarce. - **Large Sample Size:** Including over 1,500 women is a significant achievement, particularly in such a challenging and resource-limited context. - **Well-Summarized Findings:** The sections summarizing the mental health impact of conflict (e.g., Lines 71–79) are clear and concise. | As mentioned above, we are so happy for your positive appreciation, which encourages the team to work hard in the area. |  |
| Minor Corrections and Suggestions: |  |  |
| 1. **Line 57:**    - **Original:** “can be all too familiar”    - **Suggestion:** Rephrase as “can be all too common” for clarity. | Well appreciated and corrected accordingly!  [but erased due to other comments] | Line 54 |
| 2. Line 61:   - **Clarification:** “Depression, the typical psychological condition” - **Suggestion:** Replace “typical” with “most common” to avoid ambiguity. | Well appreciated and corrected accordingly  [but erased due to other comments] | Line 61 is totally omitted |
| 3. Line 80:   - **Improvement:** Add a better transition to the next paragraph. Suggested phrasing:   *“On top of these mental health stressors in developing countries, wartime has a severe negative impact on mental health, particularly for women.”* | It is well appreciated and corrected accordingly | Lines 69-70. |
| 4. Line 98 Onwards (Impact on Child Rearing):   - Consider elaborating on the impact of maternal mental disorders on children. For instance:   *“Depression or PTSD may hinder a mother’s ability to interact with her child, potentially leading to neglect or even violent behavior, which can perpetuate trauma transgenerational.”* | It is well accepted, and we appreciate your dimension of assessing the manuscript. It has been amended well! | Line 92-94 |
| 5. Line 111:   - Add: *“This may also support recommendations for targeted actions.”* | It is well appreciated and corrected accordingly | Lines 103. |
| 6. Addiction and Violence Cycles:  Highlight the importance of scaling up addiction treatment services (regarding spouses) in the region to break the cycle of violence and substance abuse. | We appreciate your comment, but since we didn’t touch much on substance abuse [not having a related hypothesis], it may be difficult to describe. Thus, it was not amended. | N/A |
| 7. Line 196 & 202:   - Check for consistent use of past tense throughout the manuscript. | We appreciate this correction. We want to apologize for our silly mistake, and we have corrected the errors. Thank you again. | Lines: 198, 200, 204 |
| 8. Table 1:   - While acceptable, the table could benefit from improved formatting for readability. This may, however, be addressed during the journal’s editing process. | Ok, but we have not responded, as there is no suggestion. | N/A |
| 9. Statistical Analysis:  Consider including a correction for multiple testing if applicable. | Since we used standard and validated measures of common mental illness and IPV, we did not add than describing it. Of course, if we validated it, it could have gained more strength. | N/A |
| 10. Line 339:   - **Original:** “The magnitude of common mental illness is too huge to compare with people living in stable conditions.” - **Suggestion:** Rephrase for clarity, e.g., *“The burden of common mental illnesses in post-conflict areas is significantly higher compared to populations in stable settings.”* | It is well appreciated and corrected accordingly | Line 357-358 |
| 11. Line 341 (Prevalence Data):   - Provide a comparison of the 1-year prevalence rates of depression. For example, the 1-year prevalence of depression is approximately 15%. | Depression and most common mental disorders are difficult to measure for a one-year prevalence, as they are measured (point) prevalence or the last two weeks (period). | N/A |
| 12. Line 343 (Local Prevalence):   - Include local pre-conflict prevalence data for Ethiopia, particularly Northern Ethiopia, and compare it with your prevalences. Reference other relevant studies or reports on post-conflict mental health in the region, including war trauma and GBV statistics. - Are there publications describing the war trauma experienced in the region? | This is also a strong comment. We should have done it, and now we have amended it. Thank you very much for your valuable comments.  We didn’t find any war-related publications on depression or anxiety in the area. | Lines 362-366 |
| 13. Line 389 (Trauma-Informed Care):   - Given the high prevalence of GBV and PTSD symptoms, recommend trauma-informed care and psychotherapy, including community-based approaches. - Suggest training healthcare workers and laypeople to provide trauma-sensitive support. | We have amended it in the recommendation session of the manuscript as it is part of a recommendation. | Line 421-430. |
| 14. Lines 387 and 408:   - Add survivors of GBV to the discussion of vulnerable groups alongside older and single women. | The comment was amended well in the discussion session and is well discussed, but is not included in the recommendation. | Line 398 to 405 |
| 15. Funding: You indicate that no funding was received. 18 data collectors had been employed for the study. How was that funded or did all oft hem contribute volunteering? Please indicate int he acknowledgements | Sorry, it is genuinely funded by International Food Policy Research International (IFPRI) [under USAID]. Sorry, we don’t know how we write it, but it may be due to a lack of funds for publication. | N/A |
| Conclusion: This study makes a significant contribution to understanding the mental health burden in the post-conflict setting of Northern Ethiopia, with a strong focus on women. Addressing the minor revisions noted above will further strengthen the manuscript and ensure its findings have the maximum impact. The recommendations, particularly for trauma-informed care and addiction treatment, are essential steps in addressing the ongoing mental health crisis in the region. | The authors’ group would like to thank the reviewers for such substantial and diversified invaluable comments that were invisible to the authors who have broadened the manuscript. | N/A |
